# *Helicobacter pylori* in bottled mineral water: genotyping and antimicrobial resistance properties

**DOI:** 10.1186/s12866-016-0647-1

**Published:** 2016-03-12

**Authors:** Reza Ranjbar, Faham Khamesipour, Nematollah Jonaidi-Jafari, Ebrahim Rahimi

**Affiliations:** Molecular Biology Research Center, Baqiyatallah University of Medical Sciences, Tehran, Iran; Cellular and Molecular Research Center, Sabzevar University of Medical Sciences, Sabzevar, Iran; Health Research Center, Baqiyatallah University of Medical Sciences, Tehran, Iran; Department of Food Hygiene, Faculty of Veterinary Medicine, Shahrekord Branch, Islamic Azad University, Shahrekord, Iran

**Keywords:** *Helicobacter pylori*, Genotyping, Antibiotic resistance properties, Bottled mineral water, Iran

## Abstract

**Background:**

Up to now, fecal–oral and oral–oral are the most commonly known routes for transmission of *H. pylori*, therefore, contaminated water can play an important role in transmission of *H. pylori* to humans. Genotyping using virulence markers of *H. pylori* is one of the best approaches to study the correlations between *H. pylori* isolates from different samples. The present research was carried out to study the *vacA*, *cagA*, *cagE*, *oipA*, *iceA* and *babA2* genotyping and antimicrobial resistance properties of *H. pylori* isolated from the bottled mineral water samples of Iran.

**Results:**

Of 450 samples studied, 8 samples (1.77 %) were contaminated with *H. pylori*. Brand C of bottled mineral water had the highest prevalence of *H. pylori* (3.63 %). The bottled mineral water samples of July month had the highest levels of *H. pylori*-contamination (50 %). *H. pylori* strains had the highest levels of resistance against metronidazole (62.5 %), erythromycin (62.5 %), clarithromycin (62.5 %), amoxicillin (62.5 %) and trimethoprim (62.5 %). Totally, 12.5 % of strains were resistant to more than 6 antibiotics. *VvacAs1a* (100 %), *vacAm1a* (87.5 %), *cagA* (62.5 %), *iceA1* (62.5 %), *oipA* (25 %), *babA2* (25 %) and *cagE* (37.5 %) were the most commonly detected genotypes. *M1as1a* (62.5 %), *m1as2* (37.5 %), *m2s2* (37.5 %) and S1a/cagA+/IceA2/oipA-/babA2-/cagE- (50 %) were the most commonly detected combined genotypes.

**Conclusions:**

Contaminated bottled mineral water maybe the sources of virulent and resistant strains *H. pylori*. Careful monitoring of bottled mineral water production may reduce the risk of *H. pylori* transmission into the human population.

## Background

In a day, Millions of people use drinking water and in some areas in which the quality of tap drinking water is low, consumption of bottled mineral water is common. Bottled mineral waters are a rich sources of trace elements copper, zinc, and iron and also magnesium, fluorine, sodium, and calcium. Their consumption is routine in hotels, restaurants, airplanes and other vehicles and also medical practitioners usually prescribe these kinds of waters for patients with malnutrition. Low risk of water-borne pathogens and chemical toxins exist for bottled drinking water, increasing their consumption over the time. In keeping with this, there are some reports regarding the contamination of these kinds of water with dangerous pathogens such as *Helicobacter pylori*, *Vibrio cholera*, *Salmonella typhimurium* and *Escherichia coli* [1, 2].

*H. pylori* is a Gram negative, micro-aerophilic and flagellated bacterium which is known as a causative agent of gastric adenocarcinoma, type B gastritis and mucosa-associated lymphoid tissue lymphoma [3, 4]. In fact, *H. pylori* colonization in gastric mucosa is the primary cause of ulcers in the stomach and duodenum [4]. Documented data revealed that near 50 % of world populations have been infected with *H. pylori* [3, 5, 6]. In keeping with this, its exact routes of transmission and origin are still unknown [7]. Based on the fecal–oral and oral–oral transmission routes of the *H. pylori*, contaminated water can play an important role in spread of *H. pylori* to humans [8, 9].

Colonization and invasion of *H. pylori* to gastric mucous is dependent on a number of virulence factors. Some of the most important virulence factors of this bacterium are the vacuolating cytotoxin (*vacA*), induced by contact with the epithelium antigen (*iceA*), cytotoxin associated gene (*cag*), blood group antigen-binding adhesion (*babA*) and outer inflammatory protein (*oip*) [10–13]. The *vacA* gene is polymorphic, comprising variable signal regions (type *s1* or *s2*) and mid-regions (type *m1* or *m2*). The *s1* type is further subtyped into *s1a*, *s1b* and *s1c* subtypes and the *m1* into *m1a* and *m1b* subtypes. The *iceA* gene has two main allelic variants *iceA1* and *iceA2* but their functions are not yet clear. The *cagA* gene has been detected in the specimens taken from the severe cases of peptic ulcer [10–13]. *CagE* is 1 of 6 genes located within the pathogenicity island shown to induce secretion of chemokines, such as interleukin IL-8, and induce inflammation [14]. The *oipA* gene plays a significant role in effective colonization of bacteria into the mucosa [15]. Genotyping using these virulence markers is considered as one of the best approaches for the study of relationships between *H. pylori* isolates from different samples.

Efficient antibiotic therapy is one of the best approaches for treatment of infectious diseases transmitted through water. Water-borne *H. pylori* infections are no exception to this principle. However, therapeutic protocols have become somewhat limited because of the presence of multidrug resistant strains of this bacterium [12, 13].

Consumption of bottled mineral water is so common among Iranian people. Besides, the prevalence of *H. pylori* is considerable among Iranian sources of infection and other sites of the wold [11, 16–20]. Therefore, study the important role of contaminated water as a risk factor for transmission of *H. pylori* is essential. These important points and also lack of community studies in relation to antibiotic resistance pattern of water-borne *H. pylori*,caused us to carried out the present study in order to investigate the exact status of *vacA*, *cagA*, *iceA*, *oipA*, *cage* and *baba2* genotypes status and antibiotic resistance pattern of *H. pylori* isolates of Iranian bottled mineral water samples.

## Results

**Fig. 1:**
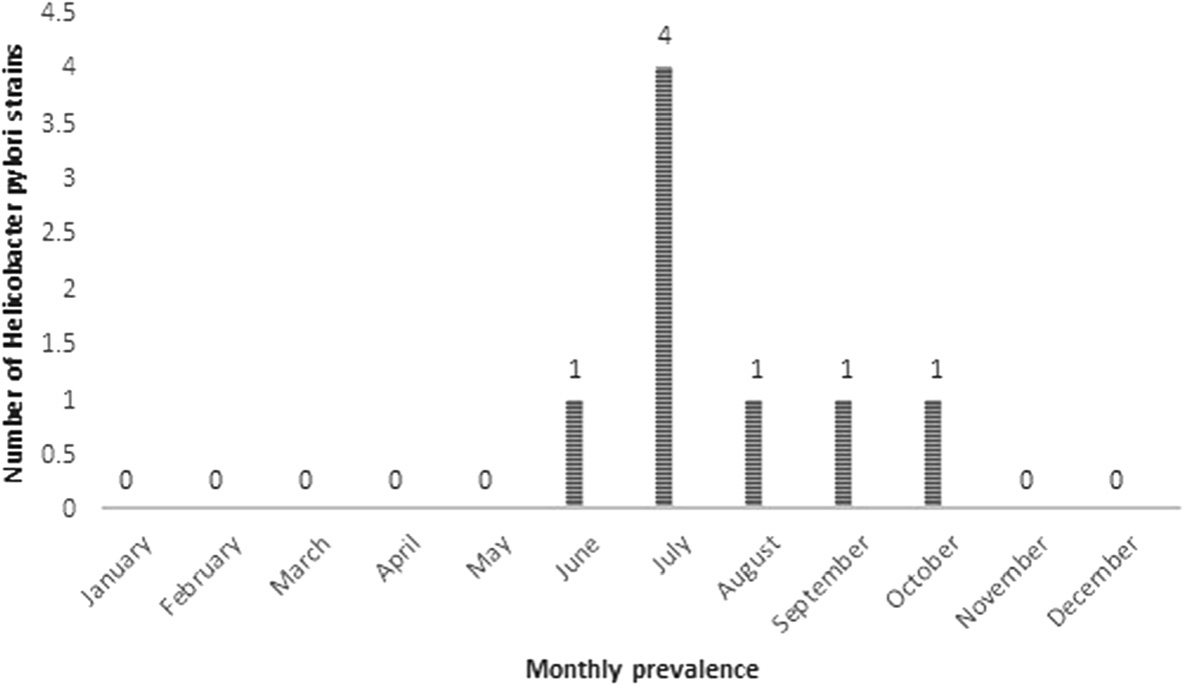
Monthly prevalence of *Helicobacter pylori* in tested bottled mineral water samples

A total of 450 bottled mineral water samples were examined for the presence of *H. pylori*, its genotypes, and its antimicrobial resistance properties. Table 1 shows the total distribution of *H. pylori* in the bottled mineral water samples of 4 different brands and various seasons from Iran. Of 450 bottled mineral water samples collected, 8 samples (1.77 %) were contaminated with *H. pylori*. The results of culture methods were confirmed using the *16S rRNA*-based PCR. The water samples of brand C had the highest prevalence (3.63 %) of *H. pylori*, while those of brand A had the lowest (0.83 %). A statistically significant difference was seen between the distributions of *H. pylori* in different brands of bottled mineral water (*P* < 0.05). We found that the samples which were collected in the summer season had the highest prevalence of bacteria (4.54 %). Significant statistical difference was seen for the prevalence of *H. pylori* between warm and cold seasons of the year (*P* < 0.05). Monthly distribution of *H. pylori* in the bottled mineral water samples is shown in Fig. 1. We found that the bottled mineral water samples from July had the highest levels of *H. pylori*-contamination (50 %).

**Table 1:** Total distribution of *Helicobacter pylori* in the tested bottled mineral water samples

Type of bottled mineral water		No. samples collected	No. *Helicobacter pylori* isolates (%)	No. isolates confirmed using *16SrRNA* based-PCR (%)
Brands	A	120	1 (0.83)^a^	1 (0.83)^a^
	B	110	1 (0.90)^a^	1 (0.90)^a^
	C	110	4 (3.63)^b^	4 (3.63)^b^
	D	110	2 (1.81)^a^	2 (1.81)^a^
	Total	450	8 (1.77)	8 (1.77)
Seasons	Spring	112	1 (0.89)^a^	1 (0.89)^a^
	Summer	110	6 (4.54)^b^	6 (4.54)^b^
	Autumn	113	1 (0.88)^a^	1 (0.88)^a^
	Winter	115	-	-
	Total	450	8 (1.77)	8 (1.77)

^a, b^Dissimilar letters in each column shows significant differences about *P* <0.05

**Fig. 2:**
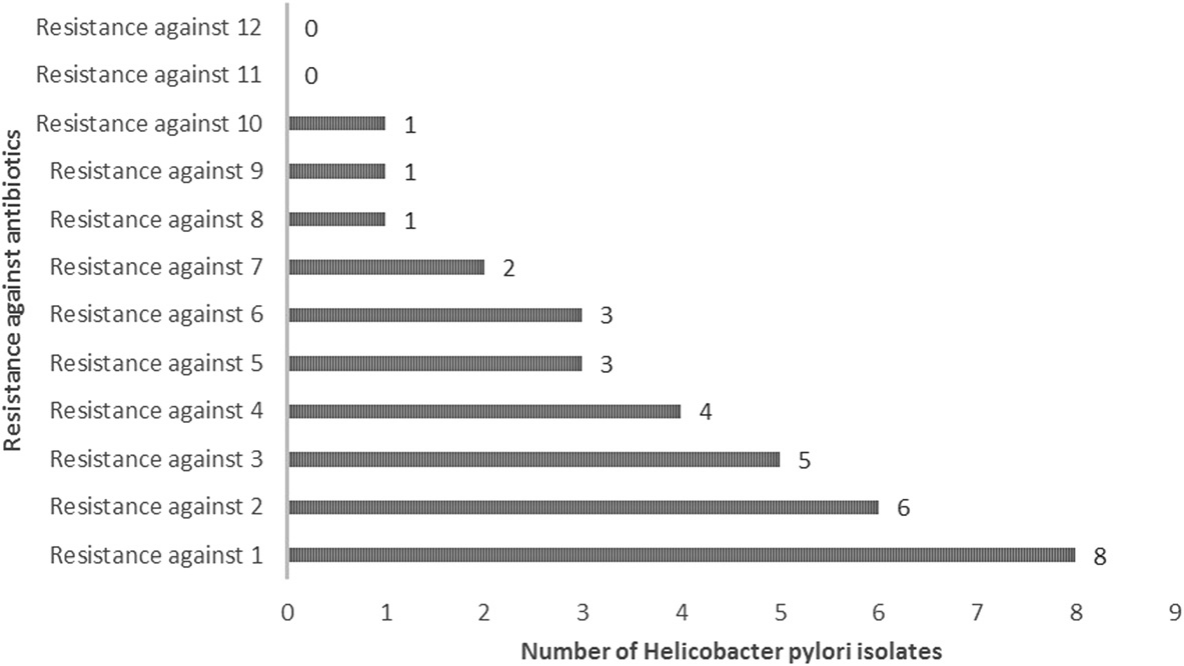
Prevalence of multidrug resistant strains of *Helicobacter pylori* isolated from tested bottled mineral water samples

The results of antimicrobial resistance pattern of *H. pylori* isolates of tested bottled mineral water samples is shown in Table 2. *H. pylori* strains of bottled mineral water harbored the highest levels of resistance against ampicillin (75 %), followed by metronidazole (62.5 %), erythromycin (62.5 %), clarithromycin (62.5 %), amoxicillin (62.5 %) and trimethoprim (62.5 %) antimicrobial agents. Prevalence of antibiotic resistance against moxifloxacin, tinidazole and ciprofloxacin were 25, 50 and 50 %, respectively. There were statistically significant differences in the levels of antibiotic resistance between ampicillin and cefsulodin (*P* =0.013), ampicillin and furazolidone (*P* =0.018), clarithromycin and furazolidone (*P* =0.029) and ampicillin and moxifloxacin (*P* =0.027). Prevalence of multidrug resistant of *H. pylori* strains of bottled mineral water samples is shown in Fig. 2. All of the 8 strains of *H. pylori* were resistant to at least one of the tested antibiotics. The highest levels of resistance was found only for one isolate which was resistant to 10 antibiotics.

**Table 2:** Antimicrobial resistance pattern of *Helicobacter pylori* isolates of tested bottled mineral water samples

Brands (No. positive)	Pattern of antibiotic resistance (%)											
	AM10^*^	Met5	ER5	CLR2	AMX 10	Lev5	Cef30	TRP25	FZL1	Mox5	Tin4	CIP5
A (1)	1 (100)^a^	1 (100)^a^	1 (100)^a^	1 (100)^a^	1 (100)^a^	-	-	-	-	-	-	-
B (1)	1 (100)^a^	1 (100)^a^	1 (100)^a^	1 (100)^a^	1 (100)^a^	1 (100)a	-	1 (100)^a^	-	-	1 (100)^a^	1 (100)^a^
C (4)	2 (50)^a^	2 (50)^a^	2 (50)^a^	2 (50)^a^	2 (50)^a^	2 (50)a	1 (25)^b^	3 (75)^a^	1 (25)^b^	1 (25)^b^	2 (50)^a^	2 (50)^a^
D (2)	2 (100)^a^	1 (50)^a^	1 (50)^a^	1 (50)^a^	1 (50)^a^	1 (50)a	1 (50)^b^	1 (50)^a^	1 (50)^b^	1 (50)^b^	1 (50)^a^	1 (50)^a^
Total (8)	6 (75)^a,^ *	5 (62.5)^a^	5 (62.5)^a^	5 (62.5)^a^	5 (62.5)^a^	4 (50)^a^	2 (25)^b^	5 (62.5)^a^	2 (25)^b^	2 (25)^b^	4 (50)^a^	4 (50)^a^

^*^AM10: ampicillin (10 μg), Met5: metronidazole (5 μg), ER5: erythromycin (5 μg), CLR2: clarithromycin (2 μg), AMX10: amoxicillin (10 μg), Lev5: levofloxacin (5 μg), Cef30: cefsulodin (30 μg), TRP25: trimethoprim (25 μg), FZL1: furazolidone (1 μg), Mox5: moxifloxacin (5 μg), Tin: tinidazole (prepared from pure powders, Sigma, 5 μg) and CIP5: ciprofloxacin (5 μg)

^a, b^Dissimilar letters in each row shows significant differences about *P* <0.05

Distribution of various genotypes of *vacA* alleles, *cagA*, *iceA1*, *iceA2*, *oipA*, *cagE* and *babA2* in the *H. pylori* isolates of bottled mineral water is shown in Table 3. The most commonly detected genotypes amongst the *H. pylori* isolates of bottled mineral water samples were *vacAs1a* (100 %), *vacAm1a* (87.5 %), *cagA* (62.5 %) and *iceA1* (62.5 %). The prevalence of *oipA*, *babA2* and *cagE* genotypes were 25, 25 and 37.5 %, respectively. The distribution of combined genotypes of *H. pylori* isolates is shown in Table 4. The most commonly detected combined genotypes were *m1as1a* (62.5 %), *m1as2* (37.5 %) and *m2s2* (37.5 %). Significant differences were found between the incidence of genotypes and brands of bottled mineral water samples (*P* <0.05). Forty seven different combined genotypes were detected in the *H. pylori* strains of bottled mineral water (Table 5). The most commonly detected combined genotypes were S1a/cagA+/IceA2/oipA-/babA2-/cagE- (50 %), M2/cagA+/IceA1/oipA-/babA2-/cagE- (37.5 %), S1a/cagA-/IceA1/oipA-/babA2-/cagE- (37.5 %), S2/cagA+/IceA1/oipA-/babA2-/cagE- (37.5 %) and M1a /cagA+/IceA2/oipA-/babA2-/cagE- (37.5 %).

**Table 3:** Total distribution of various genotypes in *Helicobacter pylori* strains of tested bottled mineral water samples

Brands (No. positive)	Distribution of various genotypes (%)												
	*S1a*	*S1b*	*S1c*	*S2*	*M1a*	*M1b*	*M2*	*CagA*	*IceA1*	*IceA2*	*OipA*	*BabA2*	*cagE*
A (1)	1 (100)	-	-	1 (100)	1 (100)	-	1 (100)	1 (100)	1 (100)	-	-	-	1 (100)
B (1)	1 (100)	-	-	-	1 (100)	-	1 (100)	1 (100)	1 (100)	-	-	1 (100)	1 (100)
C (4)	4 (100)	1 (25)	-	2 (50)	4 (100)	1 (25)	1 (25)	2 (50)	2 (50)	1 (25)	1 (25)	1 (25)	1 (25)
D (2)	2 (100)	1 (50)	-	1 (50)	1 (50)	1 (50)	1 (50)	1 (50)	1 (50)	-	1 (50)	-	-
Total (8)	8 (100)	2 (25)	-	4 (50)	7 (87.5)	2 (25)	4 (50)	5 (62.5)	5 (62.5)	1 (12.5)	2 (25)	2 (25)	3 (37.5)

**Table 4:** Distribution of combined genotypes of *Helicobacter pylori* isolated from tested bottled mineral water samples

Genotypes	Prevalence (%)^*^
M1as1a	5 (62.5)
M1as1b	1 (12.5)
M1bs1a	1 (12.5)
M1bs1b	-
M1as1c	-
M1bs1c	-
M2s1a	2 (25)
M2s1b	1 (12.5)
M2s1c	-
M2s2	3 (37.5)
M1as2	3 (37.5)
M1bs2	1 (12.5)
M1am2	2 (25)
M1bm2	-
CagA+	5 (62.5)
CagA-	3 (37.5)
CagE+	3 (37.5)
CagE-	5 (62.5)
IceA1	5 (62.5)
IceA2	1 (12.5)
IceA1 IceA2	-
OipA+	2 (25)
OipA-	6 (75)
babA2+	2 (25)
babA2-	6 (75)

^*^From a total of 8 positive strains of *H. pylori*

**Table 5:** Combined *vacA*, *cagA*, *cagE*, *iceA*, *babA2* and *oipA* genotypes of *Helicobacter pylori* isolated from tested mineral water samples

Combined genotype^*^	Frequency (%)
S1a/cagA+/IceA1/oipA+/babA2+/cagE+	1 (12.5)
S1a/cagA+/IceA1/oipA-/babA2+/cagE+	2 (25)
S1a/cagA-/IceA1/oipA-/babA2+/cagE+	1 (12.5)
S1a/cagA+/IceA1/oipA+/babA2-/cagE+	2 (25)
S1a/cagA+/IceA1/oipA+/babA2+/cagE-	1 (12.5)
S1a/cagA+/IceA1/oipA+/babA2-/cagE-	2 (25)
S1a/cagA-/IceA1/oipA-/babA2-/cagE-	3 (37.5)
S1a/cagA+/IceA1/oipA+/babA2-/cagE-	2 (25)
S1a/cagA+/IceA1/oipA-/babA2 + -/cagE-	2 (25)
S1a/cagA+/IceA2/oipA-/babA2-/cagE-	4 (50)
S1b/ cagA+/IceA1/oipA+/babA2-/cagE+	1 (12.5)
S1b/ cagA+/IceA1/oipA+/babA2+/cagE-	1 (12.5)
S1b/ cagA+/IceA1/oipA-/babA2-/cagE-	2 (25)
S2/cagA-/IceA1/oipA+/babA2+/cagE+	1 (12.5)
S2/cagA+/IceA1/oipA-/babA2+/cagE+	1 (12.5)
S2/cagA-/IceA1/oipA-/babA2+/cagE+	1 (12.5)
S2/cagA+/IceA1/oipA+/babA2-/cagE+	1 (12.5)
S2/cagA+/IceA1/oipA+/babA2+/cagE-	1 (12.5)
S2/cagA+/IceA1/oipA+/babA2-/cagE-	1 (12.5)
S2/cagA+/IceA1/oipA-/babA2+/cagE-	2 (25)
S2/cagA+/IceA1/oipA-/babA2-/cagE+	2 (25)
S2/cagA+/IceA1/oipA-/babA2-/cagE-	3 (37.5)
M1a/ cagA+/IceA1/oipA+/babA2+/cagE+	1 (12.5)
M1a/cagA-/IceA1/oipA+/babA2+/cagE+	1 (12.5)
M1a /cagA+/IceA1/oipA-/babA2+/cagE+	1 (12.5)
M1a /cagA-/IceA1/oipA-/babA2+/cagE+	1 (12.5)
M1a /cagA+/IceA1/oipA+/babA2-/cagE+	1 (12.5)
M1a /cagA+/IceA1/oipA+/babA2+/cagE-	2 (25)
M1a /cagA+/IceA1/oipA+/babA2-/cagE-	2 (25)
M1a /cagA-/IceA1/oipA-/babA2-/cagE-	2 (25)
M1a /cagA+/IceA1/oipA+/babA2+/cagE-	1 (12.5)
M1a /cagA+/IceA1/oipA-/babA2-/cagE+	2 (25)
M1a /cagA+/IceA1/oipA-/babA2+/cagE-	2 (25)
M1a /cagA+/IceA2/oipA-/babA2-/cagE-	3 (37.5)
M1b/ cagA+/IceA1/oipA-/babA2+/cagE+	1 (12.5)
M1b / cagA+/IceA1/oipA+/babA2-/cagE+	1 (12.5)
M1b / cagA+/IceA1/oipA+/babA2+/cagE-	1 (12.5)
M1b / cagA+/IceA1/oipA-/babA2-/cagE-	1 (12.5)
M2/cagA+/iceA1/oipA+/babA2+/cagE+	1 (12.5)
M2/cagA-/IceA1/oipA+/babA2+/cagE+	1 (12.5)
M2/cagA+/IceA1/oipA-/babA2+/cagE+	1 (12.5)
M2/cagA-/IceA1/oipA-/babA2+/cagE+	1 (12.5)
M2/cagA+/IceA1/oipA+/babA2-/cagE+	1 (12.5)
M2/cagA+/IceA1/oipA+/babA2+/cagE-	1 (12.5)
M2/cagA+/IceA1/oipA+/babA2-/cagE-	2 (25)
M2/cagA+/IceA1/oipA-/babA2+/cagE-	2 (25)
M2/cagA+/IceA1/oipA-/babA2-/cagE-	3 (37.5)

^*^From a total of 8 positive strains of *H. pylori*

## Discussion

The present research was carried out to study the prevalence of *H. pylori* in the bottled mineral water samples of 4 major brands in Iran as well as to determine the distribution of *vacA*, *cagA*, *cagE*, *iceA*, *oipA* and *babA2* genotype status and antibiotic resistance patterns of *H. pylori* isolates. To our best knowledge, this is the first prevalence report of genotyping and antimicrobial resistance properties of *H. pylori* in the bottled mineral water samples in the world. We found that 1.77 % of bottled mineral water samples were contaminated with resistant and virulent strains of *H. pylori*. With respect to the high accuracy which were performed on the producing of mineral water, our results reported a significant public health concern.

In contrast to findings of our investigation, Watson et al. (2004) [21] failed to isolate *H. py*lori from drinking water samples of United Kingdom. Isolation of *H. pylori* using the culture media is important due to the necessity of detecting viable bacteria in water. Previous investigation in Iran [1] showed that the distribution of *H. pylori* in various types of water samples including tap water, dental units’ water and water cooler of public places had the ranges of 2–6 %, which was higher than our results. Nurgalieva et al. (2002) [22] revealed that river water was a high risk factor for *H. pylori* infection in Kazakhstan. Accordingly, they stated that transmission of *H. pylori* could be waterborne. Drinking water can pose a substantial threat for transmission of *H. pylori* because of several important criteria [23]. These criteria include the ability of *H. pylori* to adhere to different materials and to co-aggregate with other bacteria and form complex structures on pipes or other surfaces in contact with water [23]. Notion about the disability of *H. pylori* to survive alone in running water, but to develop a symbiotic relationship and form complex structures on contact surfaces [24], makes it rational to assume that groundwater is a reservoir for *H. pylori* due to its stagnant nature. According to the high application of groundwater in the production of bottled mineral water, it is not surprising that 1.77 % of our samples were positive for this bacterium. Several investigations have shown a considerable prevalence of *H. pylori* in the ground water samples between years 1999–2007 in United Sates [25–27] and Japan [28].

Other possible reasons for the high prevalence of *H. pylori* in bottled mineral water samples in Iran are a) lack of competent approaches for water sanitization b) expending the distrustful groundwater for producing the bottled mineral water c) the opportunity of presence of bacterial colonies as a biofilm in the in pipes used for water transfer d) the opportunity of leakage of household, industrial and agricultural wastewater to the sources of mineral water and finally e) lack of personal hygiene of refinery room’s staffs. According to the high prevalence of *H. pylori* in bottled mineral water samples, it can be concluded that the food safety regulations as well as quality standards—including good manufacturing practices (GMPs), good agricultural practices (GAPs) and hazard analysis and critical control points (HACCP)—are not introduced for Iranian mineral water factories.

Marked monthly distribution with the higher prevalence in July month was seen in the *H. pylori* strains of bottled mineral water. The main reason for the higher prevalence of *H. pylori* in summer is the fact that during these times of the year climatic events including temperature, rain and barometric pressure may have influence on the prevalence of this bacterium. Bacteria have the higher growth and occurrence in warm condition. Yahaghi et al. (2014) [13] reported a similar seasonal distribution for the *H. pylori* strains of vegetable and salad samples. They showed statistically significant differences for the incidence of *H. pylori* between hot and cold seasons of the year.

Consumption of contaminated bottled mineral water of our study may lead to the gastrointestinal disorders, because of the presence of resistant and virulent strains of *H. pylori*. All of *H. pylori* isolates of our investigation were resistant to at least one antimicrobial agents. In keeping with this, 12.5 % of isolates were resistant to more than 6 antimicrobial agents. In addition, the highest levels of resistance were seen against six types of antimicrobial agents. These antimicrobial agents are the best choices for treatment of the cases of infection with *H. pylori*. In fact, in most cases of peptic ulcer diseases with the sign of *H. pylori* infection, these antimicrobial agents are used. Unfortunately, prescription of these types of antibiotics are usually irregular and over than allowance levels. Therefore, it is not surprising that majority of *H. pylori* strains were resistant to these antimicrobial agents. Similar results have been described by Bang et al. (2007) [29], Thyagarajan et al. (2003) [30], Yahaghi et al. (2014) [13] and Secka et al. (2013) [31]. High resistance levels of *H. pylori* strains of biopsy samples taken from patients with peptic ulcer diseases against metronidazole (34.7 %), clarithromycin (16.7 %) and amoxicillin (11.8 %) was reported previously by Bang et al. (2007) [29]. Another Iranian investigation [32] showed that the prevalence of resistance in *H. pylori* strains of gastrointestinal disorders against metronidazole, clarithromycin and amoxicillin had the ranges of 4 to 57 %. Bahrami et al. (2011) [33] reported that the high prevalence of water-borne *H. p*ylori resistance against furazolidone (4.5 %), clarithromycin (0.9 %), amoxicillin (0 %), metronidazole (36.4 %) and tetracycline (1.8 %). Considerable levels of resistance in *H. pylori* strains of clinical specimens against metronidazole, clarithromycin, quinolones, amoxicillin, and tetracycline have been reported from various countries such as Nigeria, Senegal, India, Saudi-Arabia, China, Colombia, Taiwan, Brazil, Thailand, Argentina and Egypt [34].

The bottled mineral water samples of our study harbored *H. pylori* positive in genotypes of *vacA*, *cagA*, *cagE*, *iceA*, *oipA* and *babA2*. The most commonly detected genotypes were *vacAs1a* (100 %), *vacAm1a* (87.5 %), *cagA* (62.5 %) and *iceA1* (62.5 %). Low prevalence of *oipA* (25 %), *babA2* (25 %) and *cagE* (37.5 %) genotypes was also detected. The number of isolates which were positive for combined genotypes was also considerable. *M1as1a* (62.5 %), *m1as2* (37.5 %) and *m2s2* (37.5 %) and also s1a/cagA+/IceA2/oipA-/babA2-/cagE- (50 %) were the most commonly detected combined genotypes. As it show, *cagA*, *oipA*, *babA2* and *cagE* negative strains of *H. pylori* were more prevalent than their positive strains. The bottled mineral water samples weren’t harbored the *cagA*, *cagE*, *babA2* and *oipa* positive strains of *H. pylori*. The number of *iceA2* strains was also low. In keeping with the uncertain cause of low distribution of *cagA*, *oipA*, *babA2* and *cagE* genotypes in the *H. pylori* strains of bottled mineral water, high prevalence of pathogenic genotypes can guarantee the occurrence of gastrointestinal disorders due to consumption of *H. pylori* positive bottled mineral water samples of our study. The number of studies which were conducted on genotyping of *H. pylori* isolated from bottled mineral water are significantly low. In an only study which was conducted by Yingzhi et al. (2002) [35], high presence of *vacAs1a*, *vacAm1a* and *vacAs1am1a* genotypes in the drinking water samples was reported. There were no additional previously published data in this field on drinking water but high presence of *vacAs1a*, *vacAm1a*, *vacAs2* alleles and also *m1as1a*, *m1as2*, *m1as1b*, *m1bs1b*, *m2s1a*, *m2s2* and *m1am2* genotypes in the *H. pylori* strains of human clinical samples such as gastric biopsy, feces and saliva have been reported previously [36–38]. Sedaghat et al. (2014) [39] reported that the prevalence of *cagA*, *iceA1*, *iceA2*, *oipA* and *babA2* genotypes in the cases of human clinical disorders including gastric ulcer and gastritis were 62.2, 48.6, 16.2, 81.1 and 94.6 %, respectively which was similar to our finding in bottled mineral water. High potential of *vacA* positive strains of *H. pylori* for causing human clinical disorders has been reported previously [11–13, 40]. The severity of diseases caused by *babA* positive strains of *H. pylori* is higher than *babA* negative strains [41]. We found that 25 % of *H. pylori* strains recovered from the bottled mineral water samples harbored the *babA* genotype. The expression of *iceA1* is up-regulated on contact between *H. pylori* and human epithelial cells and may be related with peptic ulcer disease. We found that 62.5 % of our strains were *iceA1* +. The expression of the *oipA* associated with IL-8 induction and is related with severe clinical outcomes [11–13, 40]. Prevalence of this genotype in the *H. pylori* strains of our investigation were 25 %.

## Conclusions

To our best knowledge, the present investigation is the first prevalence report for presence of *H. pylori* in bottled mineral water as well as its genotyping and antimicrobial resistance properties all-around the world. Bottled mineral water samples of Iranian factories harbored virulent and resistant strains of *H. pylori* which shows an important public health problem. Brand C, July month, resistance against ampicillin, *s1a* allele, *m1as1a* genotype and s1a/cagA+/IceA2/oipA-/babA2-/cagE combined genotypes were the most commonly detected characters of *H. pylori* isolates of bottled mineral water samples of our study. Cefsulodin and furazolidone prescription can be effective for treatment of cases of *H. pylori*.

## Methods

### Sample collection

According to the prevalence of *H. pylori* in water samples of some previous studies, the number of samples was calculated based on the following formula:

$$ \mathrm{N}={\mathrm{z}}^2\left({\mathrm{pq}/\mathrm{d}}^2\right) $$

From April 2014 to April 2015, overall 450 bottled mineral water samples of various brands (A-D) were randomly collected from the Isfahan province, Iran. Bottled mineral water samples were collected based on the protocols presented by International Standard Organization (ISO 5667-1:1980, ISO 5667-2:1991 and ISO 5667-4:1987). Samples (100 mL) were transported to the lab on ice, and used within 2 h of collection.

### Isolation of Helicobacter pylori

Samples were filtered through 0.045 μm filter membranes (Albet Co.). Each membrane was then immersed into 2 mL of Tryptic Soy Broth (TSB, Merck) for 1 h. After that, each 2 mL TSB was taken and cultured for *H. pylori*. Samples were cultured on Brucella agar (Merck, Germany) containing campylobacter selective supplement (5 mg/L, Merck), trimethoprim (0.25 mg/L), colistin methanesulfonate (30 mg/L), cycloheximide (100 mg/L), nalidixic acid (30 mg/L), trimethoprim (30 mg/L), vancomycin (10 mg/L) (Sigma, St. Louis, MO, USA), amphotericin B, sheep blood (5 %), and 7 % fetal calf serum (Sigma). After 72 h incubation at 37 °C in microaerophilic condition (85 % N_2_, 10 % CO_2_ and 5 % O_2_) using MART system (Anoxamat, Lichtenvoorde, The Netherlands), the bacterial growth was tested and confirmed as *H. pylori* using the gram staining, urease, and oxidase tests.

### Antimicrobial susceptibility testing

Pure cultures of *H. pylori* were applied for antibiotic susceptibility test. One strain from each *H. pylori*-positive sample was selected for this aim. Antimicrobial susceptibility test was accomplished by the Kirby-Bauer disc diffusion method using Mueller-Hinton agar (Merck, Germany) supplemented with 5 % defibrinated sheep blood and 7 % fetal calf serum, according to the Clinical Laboratory Standards Institute (CLSI 2012) [42]. The antimicrobial resistance of *H. pylori* was measured against the widely used antibiotics in cases of *H. pylori* gastric ulcer. The following antimicrobial disks (HiMedia Laboratories, Mumbai, India) were used: ampicillin (10 μg), metronidazole (5 μg), erythromycin (5 μg), clarithromycin (2 μg), amoxicillin (10 μg), levofloxacin (5 μg), cefsulodin (30 μg), trimethoprim (25 μg), furazolidone (1 μg), moxifloxacin (5 μg), tinidazole (prepared from pure powders, Sigma, 5 μg) and ciprofloxacin (5 μg). After incubation at 37 °C for 48 h in a microaerophilic atmosphere (85 % N_2_, 10 % CO2 and 5 % O_2_,), the susceptibility of the *H. pylori* was measured against each antimicrobial agents. Results were construed in accordance with interpretive criteria provided by CLSI (2012) [42]. The *H. pylori* ATCC 43504 was used a quality control organism in antimicrobial susceptibility determination.

### DNA extraction and Helicobacter pylori 16S rRNA gene amplification

Suspected colonies were also identified as *H. pylori* based on the PCR technique. Genomic DNA was extracted from the colonies with typical characters of *H. pylori* using a DNA extraction kit for cells and tissues (Roche Applied Science, Germany, 11814770001) according to the manufacturer’s instructions and its density was assessed by optic densitometry. Extracted DNA was amplified for the 16S *rRNA* gene (primers: HP-F: 5'-CTGGAGAGACTAAGCCCTCC-3' and HP-R: 5'-ATTACTGACGCTGATTGTGC-3') [43]. PCR reactions were performed in a final volume of 50 μL containing 5 μL 10 × buffer + MgCl_2_, 2 mM dNTP, 2 unit Taq DNA polymerase, 100 ng genomic DNA as a template, and 25 picomole of each primer. PCR was performed using a thermal cycler (Eppendorf Co., Germany) under the following conditions: an initial denaturation for 2 min at 94 °C; 30 cycles of 95 °C for 30 s, 60 °C for 30 s, and 72 °C for 30 s and a final extension at 72 °C for 8 min.

### Genotyping of vacA, cagA, cagE, iceA, babA2 and oipA genotypes in the Helicobacter pylori isolates of drinking water

Presence of the *iceA1*, *iceA2. oipA*, *cagA*, *cagE*, *babA2* genotypes and also various genotypes of *vacA* alleles (*s1a*, *s1b*, *s1c*, *m1a*, *m1b* and *m2*) were determined using PCR technique. List of primers and PCR program are shown in Table 6 [44–51]. PCR amplifications were performed in a programmable thermal cycler (Master Cycle Gradiant, Eppendorf, Germany) and all runs included one negative DNA control consisting of PCR grade water and two or more positive controls (26695, J99, SS1, Tx30, 88–23 and 84–183).

**Table 6:** Oligonucleotide primers and PCR conditions used for genotyping of *Helicobacter pylori* strains isolated from Iranian bottled mineral water

Genes	Primer Sequence (5’-3’)	Size of product (bp)	Volume of PCR reaction (50 μl)	PCR programs
*vacA s*_*1*_ *a*	F: CTCTCGCTTTAGTAGGAGC	213	5 μL PCR buffer 10X	1 cycle: 95 °C ------------ 1 min.
	R: CTGCTTGAATGCGCCAAAC			
*vacA s*_*1*_ *b*	F: AGCGCCATACCGCAAGAG	187	1.5 mM Mgcl_2_	32 cycle: 95 °C ------------ 45 s
	CTGCTTGAATGCGCCAAAC		200 μM dNTP (Fermentas)	
*vacA s*_*1*_ *c*	F: CTCTCGCTTTAGTGGGGYT	213		64 °C ------------ 50 s
	R: CTGCTTGAATGCGCCAAAC		0.5 μM of each primers F & R	
*vacA s*_*2*_	F: GCTAACACGCCAAATGATCC	199		72 °C ------------ 70 s
	R: CTGCTTGAATGCGCCAAAC		1.25 U Taq DNA polymerase (Fermentas)	
*vacA m*_*1*_ *A*	F: GGTCAAAATGCGGTCATGG	290		1 cycle: 72 °C ------------ 5 min
	R: CCATTGGTACCTGTAGAAAC			
*vacA m*_*1*_ *B*	F: GGCCCCAATGCAGTCATGGA	291	2.5 μL DNA template	
	R: GCTGTTAGTGCCTAAAGAAGCAT			
*vacA m*_*2*_	F: GGAGCCCCAGGAAACATTG	352		
	R: CATAACTAGCGCCTTGCA			
*cag A*	F: GATAACAGCCAAGCTTTTGAGG	300	5 μL PCR buffer 10X	1 cycle: 94 °C ------------ 1 min.
	R: CTGCAAAAGATTGTTTGGCAGA			
			2 mM Mgcl_2_	32 cycle: 95 °C ------------ 60 s
			150 μM dNTP (Fermentas)	
			0.75 μM of each primers F & R	56 °C ------------ 60 s
			1.5 U Taq DNA polymerase (Fermentas)	72 °C ------------ 60 s
				1 cycle: 72 °C ------------ 10 min
			3 μL DNA template	
*iceA*_*1*_	F: GTGTTTTTAACCAAAGTATC	247	5 μL PCR buffer 10X	1 cycle: 94 °C ------------ 1 min.
	R: CTATAGCCASTYTCTTTGCA			
*iceA*_*2*_	F: GTTGGGTATATCACAATTTAT	229/334	2 mM Mgcl_2_	32 cycle: 94 °C ------------ 60 s
	R: TTRCCCTATTTTCTAGTAGGT		200 μM dNTP (Fermentas)	
				56 °C ------------ 60 s
			0.5 μM of each primers F & R	
				72 °C ------------ 60 s
			1.5 U Taq DNA polymerase (Fermentas)	
				1 cycle: 72 °C ------------ 8 min
			5 μL DNA template	
*oip A*	F: GTTTTTGATGCATGGGATTT	401	5 μL PCR buffer 10X	1 cycle: 94 °C ------------ 2 min.
	R: GTGCATCTCTTATGGCTTT			
			2.5 mM Mgcl_2_	32 cycle: 94 °C ------------ 60 s
			200 μM dNTP (Fermentas)	
				56 °C ------------ 60 s
			0.5 μM of each primers F & R	
				72 °C ------------ 60 s
			2 U Taq DNA polymerase (Fermentas)	
				1 cycle: 72 °C ------------ 10 min
			3 μL DNA template	
*cagE*	F: TTGAAAACTTCAAGGATAGGATAGAGC R: GCCTAGCGTAATATCACCATTACCC	508	5 μL PCR buffer 10X	1 cycle: 94 °C ------------ 1 min.
			2 mM Mgcl_2_	35 cycle: 94 °C ------------ 60 s
			150 μM dNTP (Fermentas)	
				53 °C ------------ 45 s
			0.75 μM of each primers F & R	
				72 °C ------------ 45 min
			1.5 U Taq DNA polymerase (Fermentas)	
				1 cycle: 72 °C ------------ 8 min
			3 μL DNA template	
*BabA2*	F: CCAAACGAAACAAAAAGCGT	271	5 μL PCR buffer 10X	1 cycle: 95 °C ------------ 1 min.
	R: GCTTGTGTAAAAGCCGTCGT			
			1.5 mM Mgcl_2_	30 cycle: 91 °C ------------ 60 s
			200 μM dNTP (Fermentas)	
				45 °C ------------ 60 s
			0.5 μM of each primers F & R	
				72 °C ------------ 60 s
			1.25 U Taq DNA polymerase (Fermentas)	
				1 cycle: 72 °C ------------ 8 min
			2.5 μL DNA template	

### Gel electrophoresis

The PCR amplification products (10 μl) were subjected to electrophoresis in a 2.5 % agarose gel in 1X TBE buffer at 80 V for 30 min, stained with ethidium bromide, and images were obtained in a UVIdoc gel documentation systems (UK). The PCR products were identified by 100 bp DNA size marker (Fermentas, Germany) [52].

### Statistical analysis

Data were transferred to Microsoft Excel spreadsheets (Microsoft Corp., Redmond, WA, USA) for analysis. Using SPSS 16.0 statistical software (SPSS Inc., Chicago, IL, USA), Chi–square test and Fisher’s exact two-tailed test analysis was performed and differences were considered significant at values of *P* <0.05. Distribution of *H. pylori* genotypes isolated from bottled mineral water were statistically analyzed.

## Abbreviations

AM: Ampicillin
AMX: Amoxicillin
*babA*: Blood group antigen-binding adhesion
*cag*: Cytotoxin associated gene
Cef: Cefsulodin
CLR: Clarithromycin
CLSI: Clinical Laboratory Standards Institute
ER: Erythromycin
FZL: Furazolidone
*H. pylori*: *Helicobacter pylori*
*iceA*: Induced by contact with the epithelium antigen
Lev: Levofloxacin
Met: Metronidazole
*oip*: Outer inflammatory protein
PCR: Polymerase chain reaction
TRP: Trimethoprim
*vacA*: Vacuolating cytotoxin
